# Effects of Age, Cognition, and Neural Encoding on the Perception of Temporal Speech Cues

**DOI:** 10.3389/fnins.2019.00749

**Published:** 2019-07-19

**Authors:** Lindsey Roque, Hanin Karawani, Sandra Gordon-Salant, Samira Anderson

**Affiliations:** ^1^Department of Hearing and Speech Sciences, University of Maryland, College Park, College Park, MD, United States; ^2^Department of Communication Sciences and Disorders, University of Haifa, Haifa, Israel

**Keywords:** aging, temporal processing, speech perception, cognition, frequency-following response, cortical auditory-evoked potentials

## Abstract

Older adults commonly report difficulty understanding speech, particularly in adverse listening environments. These communication difficulties may exist in the absence of peripheral hearing loss. Older adults, both with normal hearing and with hearing loss, demonstrate temporal processing deficits that affect speech perception. The purpose of the present study is to investigate aging, cognition, and neural processing factors that may lead to deficits on perceptual tasks that rely on phoneme identification based on a temporal cue – vowel duration. A better understanding of the neural and cognitive impairments underlying temporal processing deficits could lead to more focused aural rehabilitation for improved speech understanding for older adults. This investigation was conducted in younger (YNH) and older normal-hearing (ONH) participants who completed three measures of cognitive functioning known to decline with age: working memory, processing speed, and inhibitory control. To evaluate perceptual and neural processing of auditory temporal contrasts, identification functions for the contrasting word-pair WHEAT and WEED were obtained on a nine-step continuum of vowel duration, and frequency-following responses (FFRs) and cortical auditory-evoked potentials (CAEPs) were recorded to the two endpoints of the continuum. Multiple linear regression analyses were conducted to determine the cognitive, peripheral, and/or central mechanisms that may contribute to perceptual performance. YNH participants demonstrated higher cognitive functioning on all three measures compared to ONH participants. The slope of the identification function was steeper in YNH than in ONH participants, suggesting a clearer distinction between the contrasting words in the YNH participants. FFRs revealed better response waveform morphology and more robust phase-locking in YNH compared to ONH participants. ONH participants also exhibited earlier latencies for CAEP components compared to the YNH participants. Linear regression analyses revealed that cortical processing significantly contributed to the variance in perceptual performance in the WHEAT/WEED identification functions. These results suggest that reduced neural precision contributes to age-related speech perception difficulties that arise from temporal processing deficits.

## Introduction

Older adults often report difficulty understanding speech, particularly in adverse listening environments (CHABA, 1988). Such difficulty could be attributed to numerous listener factors associated with the natural aging process, including age-related hearing loss (Dubno et al., 1984; Helfer and Wilber, 1990), cognitive decline (McClearn et al., 1997; Lin, 2011; Lin et al., 2013) and reduced auditory temporal processing (Schneider et al., 1994; Pichora-Fuller and Singh, 2006). Previous studies have focused on peripheral hearing loss, and ensuing loss of frequency selectivity (Florentine et al., 1980), as a primary mechanism for older adults’ speech understanding difficulties (Dubno et al., 1984; Koeritzer et al., 2018). Older adults with normal hearing, however, report similar difficulties understanding speech that may be attributed to temporal processing deficits as well as spectral deficits (Matschke, 1990). Füllgrabe et al. (2015) investigated the interplay and relative contributions of aging, cognition, and temporal processing on speech processing in younger and older normal-hearing (ONH) adults and found that sensitivity to temporal cues and cognitive ability were related to speech-in-noise identification scores. The present study aims to expand their research by including neural processing measures in a model that compares peripheral, central (midbrain and cortical processing) and cognitive contributions to perceptual performance on a perceptual task that relies on phoneme identification based on a temporal cue. In the following paragraphs, a brief overview of the role of cognition, temporal processing, and central processing in age-related speech perception deficits will be provided.

Aging affects multiple cognitive processes important for speech understanding, including working memory, processing speed and inhibitory control, which may contribute to reductions in speech understanding in older adults (Burke, 1997; Hedden and Gabrieli, 2004). Working memory is a higher-level cognitive process involving the temporary storage and processing of a limited amount of information, which is then either discarded or converted to long-term memory (Lunner, 2003; Lunner and Sundewall-Thoren, 2007). Individuals with limited working memory capacity have reduced speech recognition performance, possibly due to reduced ability to “fill in gaps” when parts of speech are inaudible or misunderstood (Lunner and Sundewall-Thoren, 2007; Gordon-Salant and Cole, 2016; Johns et al., 2018). Like working memory, reductions in speed of information processing may hinder speech perception, especially for artificially speeded (i.e., time-compressed) speech (Wingfield, 1996; Gordon-Salant and Fitzgibbons, 2001). Accuracy of speech recognition, especially in noise, is influenced by working memory capacity whereas speed of recognition is influenced by processing speed (Daneman and Hannon, 2007; Ronnberg et al., 2008, 2013; Genova et al., 2012). Inhibitory control is an individual’s ability to disregard irrelevant stimuli in the presence of relevant incoming stimuli (Pichora-Fuller and Singh, 2006). Older adults also experience greater difficulty understanding words while simultaneously ignoring irrelevant or asynchronous stimuli presented through both auditory and visual media, thus demonstrating reduced inhibitory control compared to young adults (Dey and Sommers, 2015; Cohen and Gordon-Salant, 2017; Gordon-Salant et al., 2017). It is theorized that processing a degraded acoustic signal (as would occur with reduced audibility and/or imprecise auditory temporal processing) forces older adults to rely on cognition for speech understanding (Pichora-Fuller et al., 1995; Wingfield and Grossman, 2006). If so, an interplay between older adults’ degraded auditory temporal processing and cognitive decline may exist and further exacerbate their speech perception difficulties.

Age-related degradation in auditory temporal processing may also contribute to older adults’ difficulty understanding speech. Speech signals in everyday listening situations (i.e., rapid speech, reverberant environments, noisy environments) are characterized by temporal alterations relative to “clean” speech (Gordon-Salant and Fitzgibbons, 1993; Gordon-Salant et al., 2010). For example, older adults seem to use temporal cues less effectively than do young adults in distinguishing between contrasting word-pairs that differ on the basis of duration cues. Older adults require longer intervals of silence preceding the final fricative to differentiate DISH from DITCH compared to younger adults (Gordon-Salant et al., 2006; Roque et al., 2019). Poorer duration discrimination in older versus younger adults has been demonstrated for relatively simple stimuli (i.e., tone bursts) and more complex signals (i.e., silent gaps embedded in tonal sequences) (Fitzgibbons and Gordon-Salant, 1995). This poorer performance in older adults may arise from reduced temporal precision secondary to physiological changes throughout the central auditory system, even in the presence of normal audiometric thresholds (Anderson et al., 2012; Presacco et al., 2016).

Electrophysiological measurements of auditory brainstem and cortex can be used to examine the neurophysiological mechanisms underlying age-related reductions in auditory temporal processing as manifest on behavioral tasks. The frequency-following response (FFR) is a measure that primarily arises from the inferior colliculus (IC) for stimulus frequencies greater than 100 Hz and reflects the temporal and spectral characteristics of a presented stimulus (Moushegian et al., 1973; Smith et al., 1975; Bidelman, 2018). Because the FFR provides an indirect measure of neural response fidelity of the IC, it may provide a non-invasive means of revealing aging deficits that have previously been demonstrated in single-neuron studies in the IC. For example, using an aging-mouse model, Walton et al. (1998) found that older mice had fewer IC neurons that fired in response to short-duration gaps than did younger mice. In humans, the FFR has previously revealed reduced neural synchronization to speech and non-speech stimuli in older compared to younger adults, which may lead to disruptions in phase locking to presented auditory stimuli (Clinard et al., 2010; Presacco et al., 2016; Roque et al., 2019). Previous electrophysiological studies have demonstrated that older adults exhibit decreased encoding of sustained components of presented stimuli compared to dynamic components. For example, Presacco et al. (2015) recorded FFRs to 170-ms speech syllables /da/ and /a/ and observed that neural firing in response to the /a/ syllable (as represented by response amplitude) significantly decreased after approximately 110 ms in older adults, but this drop in amplitude was not observed in younger adults. Interestingly, no such difference was noted for the /da/ syllable, which contained a 60-ms transition. This inability of older adults to sustain neural firing suggests that reduced neural synchrony secondary to loss of auditory nerve fibers may contribute to age-related response decay (Schmiedt et al., 1996; Walton et al., 1998; Presacco et al., 2015). Presacco et al. (2015) recorded responses to synthesized stimuli; the present study will expand on the original study to determine if response decay is present in older adults for vowels in naturally produced words.

Cortical auditory-evoked potentials (CAEPs) can be used to examine age-related reductions in neural synchronization at the level of apical dendrites of pyramidal neurons located within auditory cortex (Tan et al., 2004; Kerr et al., 2008). Diminished efficiency of post-synaptic GABA neurotransmission in the ascending auditory pathway may contribute to age-related reductions in inhibitory neurotransmission in primary auditory cortex (Caspary et al., 2008). Reduced inhibitory neurotransmission may impede older individuals’ auditory temporal processing, as observed in delayed cortical firing and CAEP latencies in older rats compared to younger rats (Juarez-Salinas et al., 2010). These age-related delays in cortical peak latencies have also been observed in human models (Tremblay et al., 2003; Maamor and Billings, 2017; Roque et al., 2019). The stimulus-locked activity recorded in the CAEP may consequently provide insight as to the cortical mechanisms underlying the timing and efficiency of speech processing.

The purpose of the present study is to investigate the interacting effects of aging, cognition, and neural encoding on the ability to identify phonemes based on vowel duration. To accomplish this objective, the same stimuli were used for both behavioral measures and FFR and CAEP electrophysiological measures. It was hypothesized that age-related temporal processing deficits, particularly a loss of neural synchrony to a sustained vowel, would hinder older adults’ ability to discriminate between the contrasting word pair WHEAT and WEED. Specifically, it was posited that (1) reduced temporal precision would be reflected in older adults’ reduced phase locking and poorer morphology in the FFR to a sustained vowel and in their prolonged peak latencies in the CAEP, relative to those of younger adults, (2) that cognitive performance, specifically processing speed, would correlate with precision of neural encoding and behavioral performance, and (3) that neural encoding, working memory, speed of information processing, and/or inhibitory control would contribute to the variance in speech perception based on a vowel duration contrast. A better understanding of the neural deficits underlying older adults’ perception of temporal speech cues could lead to more focused aural rehabilitation for improved speech understanding and increased socialization among the aging population, including those with normal peripheral hearing.

## Materials and Methods

### Participants

**FIGURE 1:**
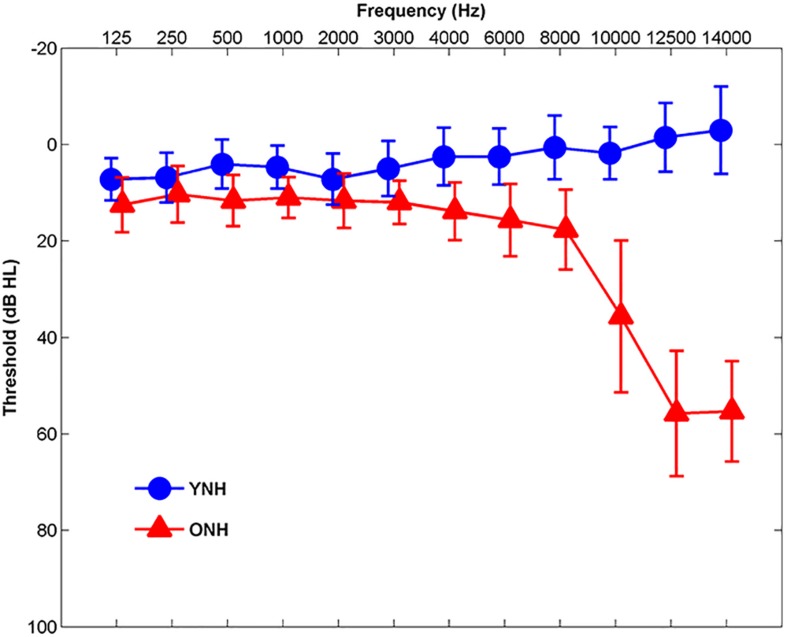
Mean audiometric thresholds of younger normal-hearing (YNH, blue) and older normal-hearing (ONH, red) participants from 125 Hz to 14,000 Hz. Clinically normal hearing was defined as pure-tone thresholds ≤20 dB HL at octave frequencies from 125 to 4000 Hz and ≤30 dB HL from 6000 to 8000 Hz. Error bars: ± 1 standard deviation.

Participants comprised younger normal-hearing (YNH, *n* = 30, 22 Females, 18–24 years, mean age and standard deviation 21.01 ± 1.55) and ONH (*n* = 30, 22 Females, 55–76 years, mean age and standard deviation 63.78 ± 5.12) adults. Clinically normal hearing was defined as pure-tone thresholds ≤20 dB HL at octave frequencies from 125 to 4000 Hz and ≤30 dB HL at 6000 and 8000 Hz bilaterally, with no interaural asymmetries ≥15 dB HL at more than two adjacent frequencies (see Figure 1). Participants were screened with two cognitive measures: the Montreal Cognitive Assessment (MoCA; Nasreddine et al., 2005) and the Wechsler Abbreviated Scale of Intelligence (WASI; Zhu and Garcia, 1999). The screening criteria were scores ≥26 on the MoCA and IQs ≥85 on the WASI. MoCA mean scores and standard deviations were 27.83 ± 1.42 and 27.90 ± 1.40 for YNH and ONH participants, respectively. YNH and ONH participants obtained mean WASI scores and standard deviations of 108.90 ± 10.68 and 107.20 ± 15.70, respectively. There was no significant effect of age on MoCA score [*t*_(58)_ = 0.18, *p* = 0.86] or WASI score [*t*_(58)_ = 0.49, *p* = 0.63]. Inclusion criteria also included normal auditory brainstem response (ABR) wave V absolute latencies (≤6.8 ms) to click stimuli and no interaural asymmetry exceeding 0.2 ms. Participants with a history of neurological dysfunction or middle ear surgery were excluded from the study. All participants were monolingual, native English speakers recruited from the Maryland, Virginia, and Washington, DC areas. All procedures were reviewed and approved by the Institutional Review Board (IRB) at the University of Maryland, College Park. Participants provided informed consent and were compensated for their time.

### Stimuli

Test stimuli comprised the contrasting word pair WHEAT (249 ms) and WEED (311 ms) that were first described in Gordon-Salant et al. (2006). This word-pair contrast depends on the single acoustic cue of vowel duration preceding the final plosive, ranging from 93 ms (WHEAT) to 155 ms (WEED). A continuum of vowel duration was created from isolated recordings of the two natural words produced by an adult American male. The endpoint stimulus perceived as WEED was a hybrid in which the final plosive /d/ was excised and replaced with a high-amplitude release from the final burst in the naturally produced WHEAT token. The continuum of vowel duration was subsequently created by removing 7–8 ms intervals of the steady-state vocalic region of WEED until it was 93 ms (the WHEAT endpoint). All stimuli were low-pass filtered at 4000 Hz at 12 dB/octave, to minimize the possible effects of high-frequency hearing threshold differences. For the perceptual identification functions, participants were presented with all nine tokens of the WHEAT/WEED continuum of vowel duration preceding the final plosive, ranging from 93 ms (WHEAT) to 155 ms (WEED). For the electrophysiology recordings, only the two endpoints of the WHEAT/WEED continuum were presented.

### Procedures

#### Cognitive

Cognition was assessed using the National Institutes of Health (NIH) Toolbox Cognition Test Battery^1^, which comprised the following: List Sorting Working Memory Test, Pattern Comparison Processing Speed Test, and Flanker Inhibitory Control and Attention Test. All three measures were administered on an iPad tablet. An experimenter assisted the participants in completing demographic questions asked on the iPad prior to testing.

##### List sorting working memory test

The List Sorting Test comprises a sequencing task in which a series of animals and/or foods were presented auditorily in the sound field and visually on the iPad screen. The participant then sorted the presented stimuli in a series and sequenced them in size order from smallest to largest (Tulsky et al., 2014). With each correct response, an additional item was added to the series, with a maximum of seven items in a series. With an incorrect response, the participant was given a second trial with a series of equal length. Testing was discontinued when the participant accurately responded to all the series or when the participant answered incorrectly during two consecutive trials. Each participant completed two versions of the test: the “1-list” version contained only names of animals, while the “2-list” version contained the names of both animals and foods. During the “2-list” version, the participant categorized the stimuli in the series before sequencing them in size order. Responses were scored for total correct responses across the two versions (Tulsky et al., 2014).

##### Pattern comparison processing speed test

The Pattern Comparison Test is a timed task in which participants were visually presented with two images on the tablet screen and indicated whether the images were identical or not. The two images may differ in type, complexity, or number of stimuli (Weintraub et al., 2013). Responses were scored for number of correct responses completed in 90 s (Carlozzi et al., 2015).

##### Flanker inhibitory control and attention test

During the Flanker Test, the participant was visually presented with a row of arrows, with a target arrow located in the center of the row. The participant then identified the left or right orientation of the centrally located arrow while ignoring the surrounding arrows, which may be congruous or incongruous in their orientation. Participants completed 25 trials. Accuracy and response time to target arrows surrounded by incongruent arrows were recorded as measures of inhibitory control and executive attention (Zelazo et al., 2014).

#### Perceptual

Participants completed an identification task similar to that implemented in Gordon-Salant et al. (2006) using the entire WHEAT/WEED continuum. The experiment was controlled and responses recorded using MATLAB (MathWorks, version 2012a). During testing, participants were seated at a desktop computer in a sound-attenuated booth. Three boxes were displayed on the computer monitor: one that read “Begin Trial” and two boxes below that read “WHEAT” and “WEED.” Participants initiated each trial by clicking the “Begin Trial” box, so testing was self-paced. Stimuli were presented monaurally to the right ear via an ER-2 insert earphone (Etymotic Research, Elk Grove Village, IL, United States) at 75 dB SPL. Following each stimulus presentation, participants indicated whether the stimulus was perceived as WHEAT or WEED by clicking on the corresponding box on the monitor. Prior to testing, participants completed a training run using only the endpoints of the WHEAT/WEED continuum and were provided feedback following each trial. Once participants achieved 90% accuracy during the training run, they completed five experimental runs, during which feedback was not be provided. Stimuli along the WHEAT/WEED continuum were each presented in quiet a total of ten times during the experimental run.

#### Electrophysiology (EEG)

EEG recordings took place during two test sessions: FFRs were recorded during one session, and ABR and CAEP recordings occurred during the other. During the recordings, participants were seated in a reclining chair in an electrically shielded, sound-attenuated booth and watched a silent, closed-captioned movie of their choice to facilitate a relaxed but wakeful state.

##### ABR

Auditory brainstem response testing to 100-μs click stimuli was performed on all participants using the Intelligent Hearing Systems Smart EP system (Intelligent Hearing Systems, Miami, FL, United States) to verify neural integrity and to provide a measure of peripheral hearing status. Clicks were presented monaurally to each ear via ER-3A insert earphone (Intelligent Hearing Systems, Miami, FL, United States) at 80 dB SPL, using a two-channel, four-electrode (Cz active, one forehead ground electrode, two earlobe reference electrodes) vertical montage. Two sets of 2000 sweeps were obtained at a presentation rate of 21.1 Hz for each ear.

##### FFR

Frequency-following responses were recorded to the two extrema of the WHEAT-WEED continuum using the Biosemi ActiABR-200 acquisition system (Biosemi B.V., Netherlands). The WHEAT and WEED stimulus waveforms were presented monaurally to the right ear via Presentation software through an ER-1 insert earphone (Etymotic Research, Elk Grove Village, IL, United States) at 75 dB SPL using alternating polarities. FFRs were recorded with a five-electrode vertical montage (Cz active, two forehead offset CMS/DRL electrodes, two earlobe reference electrodes) at a sampling rate of 16,384 Hz. A minimum of 3000 artifact-free sweeps were obtained from each participant at a rate of 2.06 Hz for WHEAT and 1.83 Hz for WEED.

##### CAEP

Cortical auditory-evoked potentials were also recorded to the two endpoints of the WHEAT-WEED continuum presented at 75 dB SPL at a rate of 0.83 Hz, with an interstimulus interval (ISI) of 0.96 s. The Biosemi Active Two system was used to record responses at a sampling rate of 2,048 Hz via a 32-channel electrode cap with earlobe electrodes (A1 and A2) serving as references. A minimum of 500 artifact-free sweeps were obtained for each stimulus from each participant.

### Data Analysis

#### Cognitive

In the NIH Toolbox application, standard scores were obtained for each of the three subtests for each participant based on normative data, as described in Carlozzi et al. (2015). For each cognitive measure (working memory, processing speed, and inhibitory control), individual raw scores were ranked to create scaled scores. A normative transformation was then applied to the ranks to derive a standard normal distribution, which was then rescaled to have a mean of 100 and a standard deviation of 15. The individual scaled scores were averaged and subsequently re-normalized.

#### Perceptual

Identification functions were computed for each individual participant by calculating the percent identification of WHEAT responses for each step along the continuum. From each identification function, the 50% crossover point was obtained to indicate the boundary of stimulus categorization. Slope of the linear portion was also calculated to represent participant distinction between the contrasting speech tokens. The 50% perceptual crossover point was obtained from each identification function using the Wichmann and Hill (2001a,b) fitting procedure and the PSIGNIFIT software^2^. Slope values were not obtained using the PSIGNIFIT software, as it takes into account the entire identification function, and performance was equivalent between groups at the extrema of the WHEAT-WEED continuum. Slope was subsequently calculated by performing linear regression analysis on the linear portion of each identification function, which approximately fell between 20 and 80% identification of WHEAT.

#### Electrophysiology (EEG)

##### ABR

ABR data were offline bandpass filtered from 70–2000 Hz using a zero-phase, 6th order Butterworth filter. An average was taken of the total 4,000 sweeps collected for each ear. In MATLAB, an automated peak-picking algorithm identified latencies and amplitudes for Waves I, III, and V within 0.5 ms of expected peak latencies, which were based on average values obtained in Anderson et al. (2012). Peak identification was confirmed by a trained peak picker who made changes where appropriate. Wave I amplitude was calculated from each participant’s average click response to verify neural integrity and serve as a peripheral measure of auditory processing. A derived horizontal montage was used to maximize Wave I amplitude. It was observed that Wave I amplitude was not normally distributed, so a square-root transformation was applied to the data. This transformed Wave I amplitude was used in subsequent statistical analyses.

##### FFR data reduction

Recorded data were analyzed in MATLAB (MathWorks, version R2011b) after being converted into MATLAB format using the pop_biosig function from EEGLAB (Delorme and Makeig, 2004). Sweeps with amplitude in the ± 30 μV range were retained. Accepted sweeps were offline bandpass filtered from 70 to 2000 Hz using a zero-phase, 4th order Butterworth filter and averaged over a 660-ms time window in MATLAB. To maximize the response of the temporal envelope, a final average response was created by averaging sweeps of both polarities.

##### Stimulus-to-response (STR) correlation

STR examines the fidelity of participants’ response waveforms in approximating the stimulus waveforms and can be considered as a means to quantify response morphology. Stimulus envelopes were extracted and bandpass filtered with the same filter used for the response envelopes. STR *r* values were obtained in MATLAB by shifting stimulus waveforms in time relative to response waveforms until reaching a maximum correlation from 10–300 ms.

##### Phase locking factor (PLF)

PLF was calculated to assess each individual participant’s phase tracking to the stimulus temporal envelope. PLF was obtained using an identical procedure to that implemented in previous studies (Jenkins et al., 2018; Roque et al., 2019). To calculate PLF values, Morlet wavelets (Tallon-Baudry et al., 1996) were used to decompose the signal from 80 to 800 Hz. Individual PLF values were calculated for the fundamental frequency (F_0_) of the stimulus vowel /i/ (138 Hz) and averaged for each participant group. PLF values were calculated for the early (60–120 ms for both speech tokens) and late vowel regions (140–200 ms for WHEAT and 200–260 ms for WEED) to examine each participant’s ability to initiate and sustain neural firing, respectively.

##### CAEP

Accepted sweeps were offline bandpass filtered from 1 to 30 Hz using a zero-phase, 4th order Butterworth filter. Eye movements were removed from the filtered data using a regression-based electrooculography reduction method (Romero et al., 2006; Schlögl et al., 2007). A 500 to 1000-ms time window was referenced to the stimulus onset for each sweep. A final response was averaged from the first 500 artifact-free sweeps. The denoising source separation (DSS) algorithm was used to remove noise/artifact from all 32 recorded channels (Särelä and Valpola, 2005; Cheveigné and Simon, 2008; Bellier et al., 2015), and to provide a measure of overall activity that is not biased toward activity from one electrode. Amplitude and latency were calculated for each prominent component of the P1-N1-P2 complex obtained from the DSS algorithm for each participant. A MATLAB automated peak-peaking algorithm was used to identify the latencies for P1, N1, and P2 in their expected time regions and to calculate area amplitudes under the curve that correspond to the designated time regions. The expected time regions were as follows: P1 (40–90 ms), N1 (90–140 ms), and P2 (140–240 ms). These expected latency regions were determined based on the average waveform for the Cz electrode, obtained for all participants.

### Statistical Analysis

All statistics were conducted in SPSS version 23.0. Independent-samples *t* tests were performed for group comparisons on ABR Wave I amplitude, perceptual 50% crossover points, slope of the identification functions, and the NIH Toolbox Cognition Test Battery measures. Repeated-measures analyses of variance (RMANOVAs) were performed to examine between-subject effects of group (YNH vs. ONH) and within-subject effects of stimulus (WHEAT vs. WEED) on FFR variables (STR, early PLF, and late PLF) and CAEP variables (peak latency and amplitude). Within-subject effects of vowel region (early vs. late) were also examined on FFR PLF variables. Independent-samples *t* tests and paired-samples *t* tests were used to perform *post hoc* analyses when significant interactions were observed. Pearson’s correlations were performed to examine relationships among cognitive, perceptual, FFR, and CAEP measures. Linear regression analyses were performed with slope of the identification functions entered as the dependent variable. Independent variables were chosen to represent different levels of the auditory system, including contributions from peripheral (Wave I amplitude), midbrain (WEED STR), and cortical variables (WEED P1 Latency). STR was chosen to represent midbrain contributions instead of PLF because a greater effect size for group differences was demonstrated for STR. We chose WEED instead of WHEAT because we expected that aging effects would be more pronounced for a longer duration vowel (Presacco et al., 2015). Cognitive variables (working memory, processing speed, and inhibitory control) were also included as independent variables. The “Stepwise” method of hierarchical regression, an automatic procedure for selecting statistical models, was performed to avoid the bias of order entry present for other methods of linear regression (i.e., hierarchical). Residuals for normality were examined to ensure that linear regression analysis was appropriate for the data. Collinearity diagnostics were completed with satisfactory variance inflation factor (highest = 1.20) and tolerance (lowest = 0.84) scores, ruling out strong correlations between predictor variables.

## Results

### Cognitive

**FIGURE 2:**
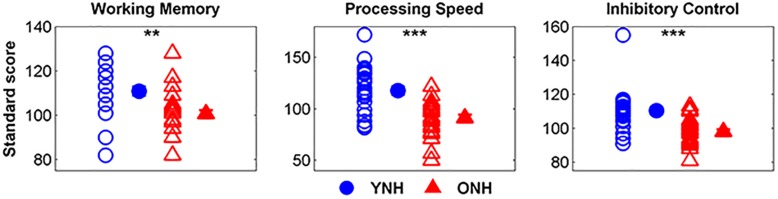
Individual and mean standard scores for younger normal-hearing (YNH, blue) and older normal-hearing (ONH, red) participants on the List Sorting Working Memory Test, Pattern Comparison Processing Speed Test, and Flanker Inhibitory Control Test. YNH participants had higher scores than ONH participants on all three subtests of the NIH Toolbox Cognition Test Battery. Error bars: ± 1 standard error. ^∗∗^*p* < 0.01, ^∗∗∗^*p* < 0.001.

Figure 2 displays mean scores and standard deviations obtained for each participant group on the three subtests of the NIH Toolbox Cognition Test Battery. We noted that 9 of 30 YNH participants demonstrated standard scores greater than two standard deviations above the mean and removed their processing speed (*n* = 8) and inhibitory control scores (*n* = 1) from group comparison and linear regression analyses. Additionally, processing speed scores for 2 of the 30 ONH participants who exhibited standard scores greater than two standard deviations below the mean were similarly excluded from further analyses. A significant effect of group was observed on working memory [*t*_(58)_ = 3.99, *p* < 0.01], processing speed, [*t*_(48)_ = 3.56, *p* < 0.01] and inhibitory control [*t*_(57)_ = 5.86, *p* < 0.001]. For each subtest, YNH participants demonstrated higher standard scores compared to ONH participants.

### Perceptual

**FIGURE 3:**
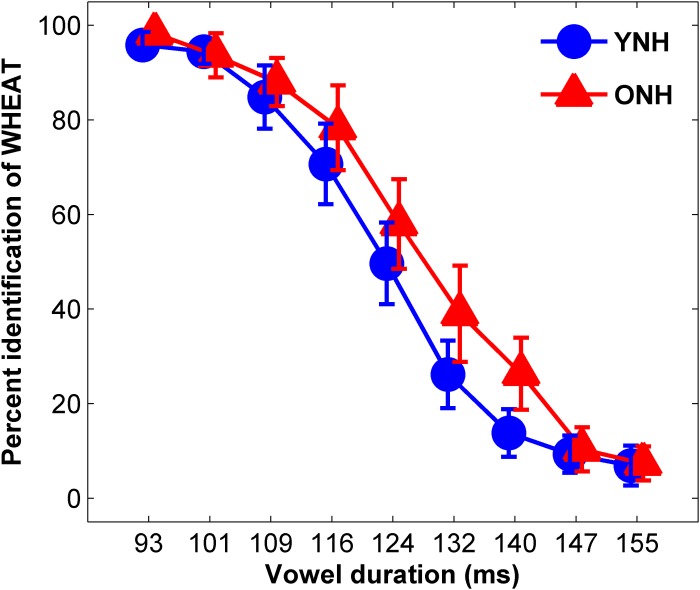
Average identification functions for percentage of trials identified as WHEAT as a function of vowel duration for each participant group. Younger normal-hearing (YNH, red) participants exhibited sharper slopes for the identification functions than did older normal-hearing (ONH, blue) participants, indicating a clearer distinction between WHEAT and WEED. Error bars: ± 1 standard error.

The average identification functions for YNH and ONH participants are displayed in Figure 3. An effect of group was observed for slope of the identification function [*t*_(58)_ = 2.49, *p* = 0.02] but not for 50% crossover point [*t*_(58)_ = 1.72, *p* = 0.09]. YNH participants demonstrated steeper slopes compared to ONH participants, indicating clearer distinction between WHEAT and WEED.

### Electrophysiology (EEG)

#### ABR

**FIGURE 4:**
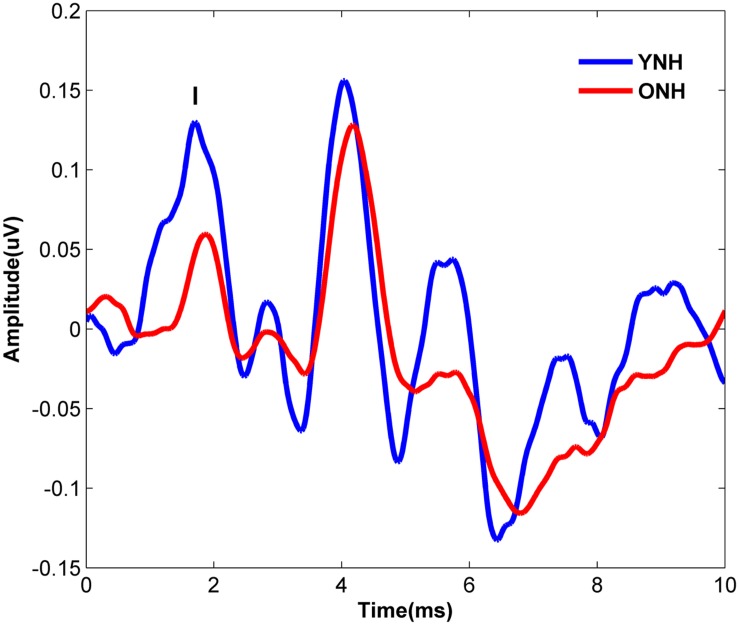
Average click-evoked ABR waveforms, derived from the horizontal electrode montage, for YNH (blue), and ONH (red) participants.

Figure 4 displays the average click-evoked ABR waveform derived from the horizontal electrode montage for each participant group. Average Wave I amplitude values were 0.38 and 0.25 μV for YNH and ONH participants, respectively. YNH participants demonstrated significantly higher Wave I amplitudes compared to ONH participants [*t*_(58)_ = 5.66, *p* < 0.001].

#### FFR

##### STR

**FIGURE 5:**
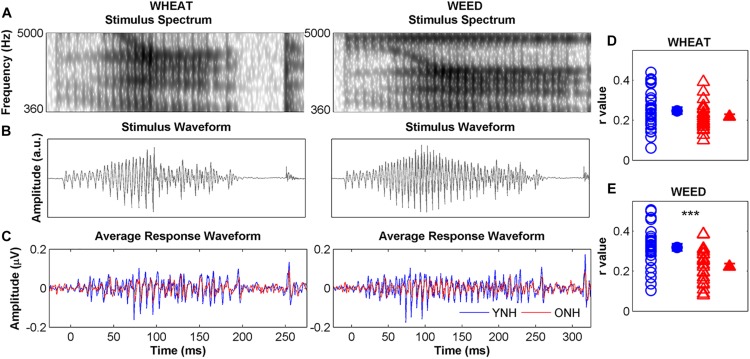
Left panel: Spectra **(A)** and waveforms **(B)** for WHEAT (93-ms vowel duration; left column), and WEED (155-ms vowel duration; right column) speech tokens. Average response waveforms **(C)** in the time domain to WHEAT and WEED for younger normal-hearing (YNH, blue) and older normal-hearing (ONH, red) participants. Right panel: Individual (open symbol) and average (closed symbol) stimulus-to-response correlation *r* values for each participant group to **(D)** WHEAT and **(E)** WEED. Response waveforms of YNH participants more closely mirrored the stimulus waveforms than did those of ONH participants. Error bars: ± 1 standard error. ^∗∗∗^*p* < 0.001.

Figure 5 compares average YNH and ONH response waveforms (panel C) to stimulus spectra (panel A), and waveforms (panel B). Individual and average STR *r* values to WHEAT and WEED are displayed in panels D and E, respectively. There were significant main effects of group [*F*(_1, 58)_ = 16.42, *p* < 0.001, $\eta_{p}^{2}$ = 0.22] and stimulus on STR [*F*_(1, 58)_ = 6.19, *p* = 0.02, $\eta_{p}^{2}$ = 0.01], as well as a significant group × stimulus interaction [*F*_(1, 58)_ = 4.74, *p* = 0.03, $\eta_{p}^{2}$ = 0.08]. YNH response waveforms better mirrored the WEED stimulus waveform than did ONH response waveforms [*t*_(58)_ = 4.18, *p* < 0.001]. However, no group difference was observed for the WHEAT stimulus waveform [*t*_(58)_ = 1.44, *p* = 0.16]. STR *r* values were higher for WEED than for WHEAT in the YNH participants [*t*_(29)_ = 2.68, *p* = 0.01] but not in the ONH participants [*t*_(29)_ = 0.32, *p* = 0.76].

##### PLF

**FIGURE 6:**
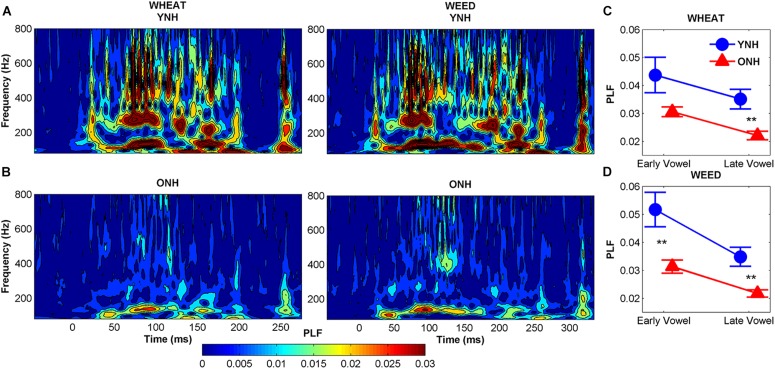
Average phase-locking factor (PLF) to the temporal envelope of WHEAT (left column) and WEED (right column) stimuli represented in the time-frequency domain, with hotter (red) colors indicating increased phase locking in younger normal-hearing (YNH) **(A)** and older normal-hearing (ONH) **(B)** participants. Right panel: Average PLF values to the 138-Hz fundamental frequency in the early and late time regions corresponding to the vowel /i/ of WHEAT **(C)** and WEED **(D)**. ONH participants demonstrated reduced phase locking compared to YNH participants. For both participant groups, phase locking declined from the early vowel region to the late vowel region to both stimuli. Error bars: ± 1 standard error. ^∗∗^*p* < 0.01.

Figure 6 compares average phase locking to the temporal envelopes of WHEAT and WEED for YNH and ONH participants. A significant main effect of group was observed, such that ONH participants demonstrated reduced phase locking compared to YNH participants [*F*_(1, 58)_ = 11.87, *p* = 0.001, $\eta_{p}^{2}$ = 0.17]. There was also a significant main effect of vowel region (early vs. late) [*F*_(1, 58)_ = 15.64, *p* < 0.001, $\eta_{p}^{2}$ = 0.21]. For both participant groups, phase locking declined from the early vowel region to the late vowel region. A significant stimulus × region interaction was observed [*F*_(1, 58)_ = 4.54, *p* = 0.04, $\eta_{p}^{2}$ = 0.07], such that the decline in phase locking from the early to late vowel region was more pronounced to WEED than to WHEAT. No other significant main effects or interactions were noted.

#### CAEP

An omnibus RMANOVA was conducted to compare differences between YNH and ONH groups for the three cortical peak (P1, N1, P2) amplitudes and latencies across both stimuli (WHEAT vs. WEED).

##### Latency

**FIGURE 7:**
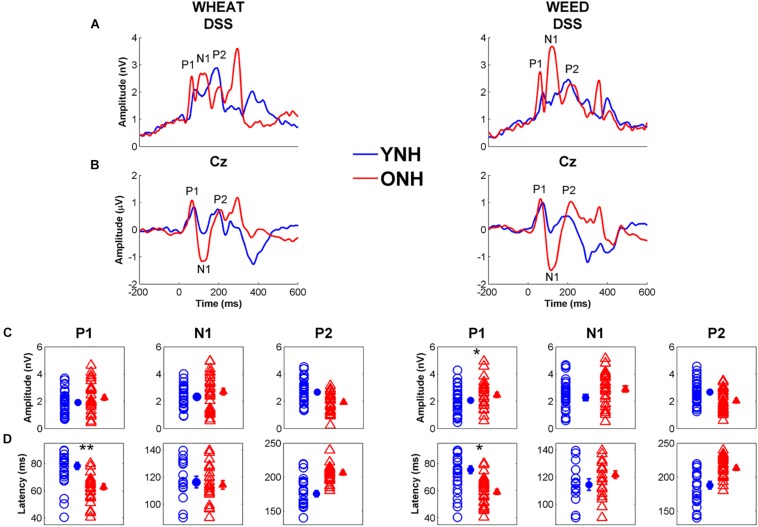
Grand average cortical auditory-evoked waveforms obtained through the denoising source separation (DSS) algorithm **(A)** and from the Cz electrode **(B)** for younger normal-hearing (YNH, blue) and older normal-hearing (ONH, red) participants. Note that the time regions for latency and amplitude analyses were based on the Cz electrode waveforms. The waveforms with Individual (open symbol) and average (closed symbol) amplitudes **(C)** and latencies **(D)** for prominent cortical peaks to WHEAT (left column) and WEED (right column) obtained from the DSS algorithm. P1 latencies were earlier in the ONH compared to YNH participants for both stimuli. Error bars: ± 1 standard error. ^*^p < 0.05, ^∗∗^*p* < 0.01.

Figure 7 displays average CAEP response waveforms obtained from the DSS analysis, as well as individual and average peak amplitudes and latencies for YNH and ONH participants. There was a significant main effect of group on CAEP peak latency [*F*_(1, 58)_ = 8.06, *p* < 0.01, $\eta_{p}^{2}$ = 0.12] and a significant peak × group interaction [*F*_(2, 57)_ = 5.08, *p* = 0.01, $\eta_{p}^{2}$ = 0.16]. RMANOVA models (within-group variable: stimulus, between-group variable: age group) were subsequently performed to examine differences for each peak individually. The ONH participants exhibited earlier peak latencies compared to YNH participants for P1 [*F*_(1, 58)_ = 33.23, *p* < 0.001, $\eta_{p}^{2}$ = 0.36]. No group difference was observed for N1 latency [*F*_(1, 58)_ = 0.12, *p* = 0.73, $\eta_{p}^{2}$ < 0.01] or P2 latency [*F*_(1, 58)_ = 1.40, *p* = 0.24, $\eta_{p}^{2}$ = 0.02]. A stimulus × peak interaction was also observed [*F*_(2, 57)_ = 3.63, *p* = 0.03, $\eta_{p}^{2}$ = 0.11], such that P1 was earlier for WHEAT than for WEED [*t*_(59)_ = 2.20, *p* = 0.03]. No other significant interactions were observed [all *p* values > 0.05].

##### Amplitude

No significant effects of group [*F*_(1, 58)_ = 2.46, *p* = 0.12, $\eta_{p}^{2}$ = 0.04] or stimulus [*F*_(1, 58)_ = 3.58, *p* = 0.06, $\eta_{p}^{2}$ = 0.06] were observed on CAEP peak amplitudes. However, there was a peak × group interaction [*F*_(2, 57)_ = 6.21, *p* = 0.004, $\eta_{p}^{2}$ = 0.18], and ANOVA models were subsequently performed for each peak individually. ONH participants exhibited larger P1 amplitudes than YNH participants [*F*_(1, 58)_ = 5.35, *p* = 0.02, $\eta_{p}^{2}$ = 0.08]. No effect of group was observed for N1 or P2 amplitude to either stimulus [all *p* values > 0.05].

### Multiple Linear Regression

Results of the multiple linear regression analyses indicated that cortical factors predicted variance in slope of the identification functions. Table 1 displays Pearson’s correlation coefficients (*r*) among the predictor variables entered in the linear regression analyses. Table 2 displays the standardized coefficients and levels of significance for the independent variables for the one model created during the Stepwise linear regression analysis. Slope of the perceptual identification functions correlated with WEED P1 Latency [*r* = −0.39, *p* < 0.01]. All three cognitive measures significantly correlated with one another [all *p* values < 0.05], with the exception of working memory and processing speed, which did not correlate [*p* = 0.32]. Wave I amplitude significantly correlated with all other variables entered into the correlation matrix [all *p* values < 0.01], except for WEED STR [*p* = 0.06] and processing speed [*p* = 0.45]. During the linear regression analysis, predictor variables sampled were Wave I amplitude, WEED STR, WEED P1 Latency, working memory, processing speed, and inhibitory control. These predictor variables were chosen due to observed group differences and to represent potential peripheral, central, and cognitive contributions. In the final model only WEED P1 Latency significantly contributed to variance in slope. This model was a good fit for the data [*F*_(1,__48)_ = 8.22, *p* < 0.01], with an *R*^2^ value of 0.15.

**TABLE 1:** Intercorrelations among slope and the independent peripheral, central, and cognitive variables.

**Variables**	**1. Slope**	**2. Wave I AMP**	**3. WEED STR**	**4. WEED P1 LAT**	**5. WM**	**6. PS**	**7. IC**
1. Slope							
2. Wave I AMP	–0.21						
3. WEED STR	0.13	0.27					
4. WEED P1 LAT	–0.39^∗∗^	0.41^∗∗^	0.02				
5. Working Memory (WM)	–0.13	0.46^∗∗^	0.10	0.18			
6. Processing Speed (PS)	–0.08	0.11	0.30^*^	0.14	0.15		
7. Inhibitory Control (IC)	–0.21	0.31^*^	0.17	0.21	0.45^∗∗^	0.58^∗∗∗^	

Results of Pearson’s correlational analysis indicated that the slope is only correlated to WEED P1 LAT. All three cognitive variables are strongly correlated with one another, except for working memory, and processing speed. Wave I amplitude correlated with all other predictor variables, except for processing speed. ^*^p < 0.05, ^∗∗^*p* < 0.01, ^∗∗∗^*p* < 0.001. AMP, amplitude; STR, stimulus-to-response correlation; LAT, latency.

**TABLE 2:** Summary of “Stepwise” regression analysis for variables contributing to slope of the perceptual identification functions.

**Variable**	***R*^2^ change**	**β**	***p* value**
Model 1	0.15		0.006
WEED P1 LAT		−0.39	0.006

*Standardized (β) coefficients in a model automatically generated by evaluating the significance of each variable’s contribution to slope of the perceptual identification functions. Only one model was generated, in which WEED P1 LAT predicts significant variance in slope. All other variables were excluded from the model (Wave I AMP, WEED STR, working memory, processing speed, and inhibitory control). STR, stimulus-to-response correlation; LAT, latency.*

## Discussion

The purpose of the current study was to investigate the interplay between cognition, perception, and neural processing of temporal speech cues to gain a better understanding of the communication difficulties often experienced by older adults with normal hearing. To accomplish this objective, we investigated the effects of age on neural temporal encoding underlying phoneme identification based on vowel duration, as well as possible cognitive contributions to variability in perceptual performance on a phoneme identification task. The data support some, but not all, of our initial hypotheses. As expected, younger adults exhibited higher cognitive functioning in the domains of working memory, speed of information processing, and inhibitory control relative to older adults. Younger adults also demonstrated sharper slopes for the perceptual identification functions than did older adults, suggesting a clearer distinction between WHEAT and WEED. Electrophysiological measurements revealed age-related deficits in neural encoding of the extrema of the WHEAT/WEED continuum of vowel duration at both the level of the auditory brainstem and cortex. FFRs revealed poorer morphology (reduced STR correlations) and reduced phase locking to the stimulus temporal envelopes (lower PLF values) in older adults compared to younger adults. In contrast to our initial hypothesis, CAEPs revealed earlier P1 latencies for older adults than for younger adults. Additionally, linear regression analyses revealed that only cortical factors significantly contributed to variance in slope of the perceptual identification functions.

### Cognitive Functioning

Consistent with previous studies, older adults demonstrated decreased cognitive functioning in the domains of working memory (Salthouse and Babcock, 1991), processing speed (Salthouse, 1996), and inhibitory control (Salthouse, 2010). For individuals above 20 years of age, validity studies of the NIH Toolbox Cognition Battery have found significant negative correlations between age and performance on the List Sorting Working Memory Test, Pattern Comparison Processing Speed Test, and Flanker Inhibitory Control and Attention Test (Weintraub et al., 2013).

### Perceptual

Although the procedure for the perceptual identification task stemmed from that implemented in Gordon-Salant et al. (2006), different patterns of results were observed between the original experiment and the current study. For the contrasting word-pair WHEAT and WEED, younger and older adults with normal hearing did not significantly differ in 50% crossover point or slope of the identification functions in Gordon-Salant et al. (2006). In the current study, performance between groups was equivalent for the 50% crossover point, but younger adults demonstrated steeper slopes than did older adults. The different result patterns may be attributed to differences in presentation level utilized in the two different studies. Gordon-Salant et al. (2006) utilized a presentation level of 85 dB SPL compared to the 75 dB SPL presentation level used in the current study. Audibility may consequently impact the clarity of older adults’ distinction between WHEAT and WEED, with softer presentation levels obscuring their ability to distinguish between the contrasting word pair.

### Peripheral Function

The click-evoked ABR was used to evaluate peripheral function, as age-related reductions in Wave I amplitude have been previously documented (Psatta and Matei, 1988; Grose et al., 2019). While the association between aging and hearing loss is believed to drive these group differences, the present study demonstrates that older adults with clinically normal hearing can also exhibit reduced Wave I amplitude compared to younger adults. It has been suggested that noise exposure may contribute to reduced Wave I amplitudes in adults who simultaneously demonstrate normal pure-tone thresholds and cochlear outer hair cell function (Konrad-Martin et al., 2012; Bramhall et al., 2017). Most of the current literature supporting this noise-induced cochlear synaptopathy, however, exists among animal models; the evidence is mixed in humans (Bharadwaj et al., 2015; Liberman et al., 2016; Grose et al., 2017). It is possible, however, that Wave I may be decreased in amplitude due to age-related losses of auditory nerve fibers and cochlear synaptopathy that are independent of noise exposure history (Schmiedt et al., 1996; Sergeyenko et al., 2013). Elevated thresholds in the extended high-frequency range in the older listeners may also be a factor in the reduced Wave I amplitudes (Verhulst et al., 2016).

### Subcortical Representation

Similar to the results observed in Roque et al. (2019), aging affected FFR response morphology. Older adults exhibited less accurate neural representations of stimulus waveforms than younger adults. Rat models have also demonstrated similar aging effects on neural representation of sinusoidally amplitude-modulated tones (Parthasarathy and Bartlett, 2011). Older rats’ response fidelity was poorer than those of younger rats, suggesting that temporal processing deficits limit the older rats’ ability to encode changing envelope shapes (as would also occur in speech). This degraded neural representation of speech stimuli in the aging midbrain may be attributed to reduced neural synchrony. Human studies have also suggested that desynchronization may inhibit older adults’ ability to encode the rapidly changing temporal and spectral properties of speech (Anderson et al., 2012; Presacco et al., 2015, 2016).

In contrast to the results found in Roque et al. (2019), however, the present study observed aging effects on phase locking to the temporal envelope of WHEAT and WEED. The difference in findings between the two studies may be attributed to the fact that phase locking was examined to a vowel region following the plosive /d/ in Roque et al. (2019), whereas the current study examined phase locking to a vowel region following a glide. Overall, reduced phase locking was observed to the glide-vowel region in WHEAT/WEED than to the plosive-vowel region in the DISH/DITCH contrast used in the Roque et al. (2019) study. This difference might be due to increased synchronous firing generated by the shorter stimulus length of the stop-constant burst in the DISH/DITCH contrast compared to that generated by the glide. Brief stimuli are most effective at generating synchronous firing (Durrant and Boston, 2007). Because deficits in temporal synchronization in the IC as shown in mice (Walton et al., 1998) may underlie older adults’ reduced phase locking, age-related reductions in neural synchrony would be exacerbated for the processing of longer-duration signals (i.e., glides) compared to shorter-duration signals (i.e., plosives).

Presacco et al. (2015) compared aging effects on the encoding of the synthetic plosive-vowel syllable /da/ and vowel /a/. Younger participants exhibited more robust FFR encoding than older participants, but these group differences were more pronounced for the sustained vowel region, especially in the last 60 ms of the /a/ vowel during which an abrupt decrease in synchronization was seen in many of the older participants. Based on these results, we had hypothesized that older participants’ phase locking would decline from the early vowel region to the late vowel region to a greater extent than in young participants due to an inability to sustain neural firing. Results of the current study, however, showed that both younger and older participants were unable to maintain phase locking over time. Neural adaptation at the levels of the auditory nerve and midbrain may limit the duration of neural firing, particularly in response to a static signal (Sumner and Palmer, 2012; Pérez-González and Malmierca, 2014), leading to a reduction in phase locking over time. Additionally, the older participants’ phase locking did not decrease over time to the same degree as the younger participants. The older participants’ phase locking was already reduced and close to the noise floor in the early vowel region; therefore, their phase locking cannot decrease to the same extent as in younger participants with sustained stimulation.

### Cortical Representation

It has been suggested that prominent CAEP components correspond to different sub-conscious processes that precede the conscious percept of an incoming stimulus. P1 and N1 are earlier-occurring peaks that reflect the pre-perceptual detection and focusing of attention to presented stimuli, respectively (Näätänen and Winkler, 1999). P2 emerges later, around 200 ms, and may reflect auditory object identification of presented stimuli (Näätänen and Winkler, 1999). Earlier and larger early peak components have been observed in older adults compared to young adults using magnetoencephalography (MEG) (Brodbeck et al., 2018). This neural activity, occurring at ∼30 ms, was source-localized to left temporal lobe, in regions lateral and inferior to auditory cortex. Brodbeck et al. (2018) suggested that this increased engagement of neural activity during speech detection may reflect increased neural excitability due to an age-related imbalance of inhibitory and excitatory processes that has been shown in animal models (Caspary et al., 1995; Hughes et al., 2010). This increased excitability may manifest as robust onset responses (i.e., larger and earlier) to a presented auditory signal (Alain et al., 2014).

In contrast to Roque et al. (2019), the present study did not observe group differences in P2 latency. The lack of difference in the current study may be due to greater low-frequency energy for the initial consonant in WHEAT/WEED (F1 starting frequency: 320 Hz, F2 starting frequency: 900 Hz) compared to that for DISH/DITCH (F1 starting frequency: 465 Hz, F2 starting frequency: 2080 Hz). Additionally, stricter audiometric criteria and lower age cutoffs were employed in the present study compared to Roque et al. (2019) for the older participants. Stimuli were also low-pass filtered at 4000 Hz to ensure audibility. Reduced audibility may affect the robustness of auditory object identification represented by the P2 peak component; therefore, these study design factors would all reduce audibility confounds for high-frequency stimuli, where we found the largest group differences. Finally, the previous studies that reported delayed P2 latencies in older compared to younger adults used different cortical analyses (Tremblay et al., 2003; Billings et al., 2015), either reporting on a single electrode (e.g., Cz) or reporting global field power, which yields the standard deviation across all electrodes over time (Skrandies, 2005). The DSS algorithm used in this study may minimize group differences by reducing noise that might be otherwise present in the older adults’ responses.

### Relationships Among Cognitive, Perceptual, and EEG Variables

Select cortical variables (peak latency) contributed to variance in perceptual performance. Cortical processing appears to be an important factor in perceptual performance in young adults (Billings et al., 2013). In older adults, Billings et al. (2015) found that N1 and P2 amplitudes and latencies predicted recognition of sentences presented at various signal-to-noise ratios. In contrast to the current study, they did not find correlations between the P1 components and behavioral performance. This difference in findings may be due to the nature of the behavioral task. Repeating sentences in noise would draw on cognitive processes to a greater extent than identifying words in quiet and may have increased engagement for the later cortical components. In our study, we included the FFR to evaluate processing at subcortical levels. The extent to which the cortex compensates for auditory degradation at earlier subcortical levels may determine successful behavioral performance. We noted that the regression analysis was driven by a correlation in the older participants (*r* = −0.39) that was not significant in the younger participants (*r* = −0.27). In the older adults, earlier latencies correlated with shallower slopes. This finding is consistent with a previous MEG study that found an increase in early activity for the more ambiguous stimuli on a perceptual identification function in young adults (Gwilliams et al., 2018). Therefore, earlier latencies (i.e., greater cortical activation) may suggest that the endpoints of the identification function are ambiguous, resulting in a shallower slope.

Although previous studies found that midbrain factors significantly contribute to the perception of temporal speech cues (Roque et al., 2019), the current study only observed neural contributions from auditory cortex. It is possible that these results diverge from those previously documented due to the fact that Roque et al. (2019) examined contributing factors to 50% crossover point, whereas this study examined contributions to slope of the perceptual identification functions. Because slope corresponds to the listeners’ subjective distinction of two words it is likely dependent on auditory object identification, which occurs at the level of the auditory cortex (Ross et al., 2013). Although precise representation of the speech signal in midbrain may impact auditory object representation in cortex, the degree to which the cortex compensates for age-related deterioration in phase locking may be the most important contributing factor to perception of temporal cues. No correlations were observed between subcortical and cortical variables in the present study when including both younger and older participants in the analysis and when performing the correlations separately for each group (all *p* values > 0.05). Bidelman et al. (2014) observed a correlation between magnitude of first formant representation in the brainstem and the CAEP N1- P2 amplitude in older adults. It should be noted that this association seems to be mediated by hearing loss. Bidelman et al. (2014) suggested that this relationship implied more redundancy along the ascending auditory system in older adults. In addition, Presacco et al. (2019) found that the reconstruction accuracy in cortex correlated with midbrain quiet-to-noise correlations in participants with hearing loss but not in participants with normal hearing. Presacco et al. (2019) suggested that hearing loss alters connectivity between midbrain and cortex, so perhaps correlations among these factors would have been observed if participants with hearing loss had been included in the present study.

Peripheral and cognitive variables did not contribute to variance in perceptual performance. The original purpose of the present study was to examine effects of aging independent of peripheral hearing loss. The stimuli used in the present study were low-pass filtered at 4000 Hz to reduce audibility confounds. Therefore, it is expected that peripheral factors would have played a larger role for unfiltered stimuli. Further, reduced auditory perception (i.e., peripheral hearing loss) may force listeners to employ cognitive processes for speech understanding (Pichora-Fuller et al., 1995; Wingfield and Grossman, 2006). We note that the perceptual identification task used in the present study was not cognitively demanding. Although the task likely employed short-term memory, we theorize that cognitive processing would have significantly contributed to variance in perceptual performance had our task employed sentence-level materials and/or speech stimuli presented in noise, all of which would increase the cognitive load required for the task and are known to be related to working memory (Akeroyd, 2008; Füllgrabe et al., 2015).

It should be noted that the present study did not employ a task that combines a cognitive task (e.g., working memory or response inhibition) with behavioral testing or EEG recording. The current study was primarily interested in the neural representation of a specific temporal speech cue, and we therefore needed to present stimuli for thousands (FFR) or hundreds (CAEP) of trials to obtain adequate noise-free recordings in the time domain. An alternate approach would be to record cortical responses during an active listening task that might task attention or memory. This approach has been used previously to document the differing effects of attention on cortical processing in younger versus older adults (Henry et al., 2017).

## Conclusion

The current study showed that neural encoding of P1 latency in the auditory cortex contributed to older adults’ less distinct perceptions of contrasting word pairs differing in vowel duration, compared to younger adults. The communication struggles resulting from reduced temporal precision may lead to older adults’ misunderstanding of spoken language and subsequent frustration, especially among those with normal hearing who often state that they can hear a talker just fine but have difficulty understanding what was said. It remains an open question as to whether auditory training can improve temporal processing (Henshaw and Ferguson, 2013). In an aging rat model, auditory training was able to partially reverse age-related declines in myelination and improve temporal processing in the auditory cortex, possibly mediated by an increase in inhibitory neurotransmission (de Villers-Sidani et al., 2010). An imbalance of inhibitory/excitatory transmission may lead to more diffuse neural firing, decreased temporal processing, and poor perception (Caspary et al., 2008). Given that decreased inhibition may mediate overrepresentation in auditory cortex, auditory training and/or pharmacologic intervention may lead to restoration of the precise temporal processing needed for the discrimination of speech stimuli.

## Ethics Statement

This study was carried out in accordance with the recommendations of the Institutional Review Board of the University of Maryland with written informed consent from all subjects. All subjects gave written informed consent in accordance with the Declaration of Helsinki. The protocol was approved by the Institutional Review Board of the University of Maryland.

## Author Contributions

SA and LR designed the experiment. SG-S and HK provided inputs into the study design and theoretical framework. LR and HK collected and analyzed the data. LR, HK, SG-S, and SA wrote the manuscript.

## Conflict of Interest Statement

The authors declare that the research was conducted in the absence of any commercial or financial relationships that could be construed as a potential conflict of interest.

## Footnotes

1 www.nihtoolbox.org

2 https://sourceforge.net/projects/psignifit/
